# Evaluation of dose linearity in the systemic availability and pharmacokinetics of topically administered diclofenac: A ^14^C-microdosing study in healthy volunteers

**DOI:** 10.1016/j.dmd.2025.100091

**Published:** 2025-05-08

**Authors:** Severin Mairinger, Mihye Kwon, Martin Bauer, Jinho Song, Edith Lackner, Anselm Jorda, Felix Bergmann, Iris K. Minichmayr, Ka Yeon Kim, Min Sun Choi, Jae Hoon Shim, Stephen R. Dueker, Markus Zeitlinger, Oliver Langer

**Affiliations:** 1 Department of Clinical Pharmacology, Medical University of Vienna, Vienna, Austria; 2 Department of Biomedical Imaging and Image-Guided Therapy, Medical University of Vienna, Vienna, Austria; 3 Korea Radio-Isotope Center for Pharmaceuticals, Korea Institute of Radiological & Medical Sciences, Seoul, Republic of Korea

**Keywords:** Dose linearity, Microdialysis, Microdosing, Topical drugs

## Abstract

An important safety consideration for topically administered drugs is the extent of systemic exposure they achieve. The aim of this study was to evaluate whether a topically administered microdose of the model drug diclofenac can predict the systemic availability, plasma, and tissue pharmacokinetics of a topical therapeutic dose. Eight healthy participants (6 men and 2 women) participated in a 4-period, crossover study. In period 1, a topical microdose (62 ± 6 *μ*g) was administered; in period 2, a single intravenous microdose (0.95 ± 0.03 *μ*g) was administered; in period 3, a topical therapeutic dose (120 mg) was administered; and in period 4, a single intravenous therapeutic dose (75 mg) of [^14^C]diclofenac was administered, with or without the addition of unlabeled diclofenac. Venous blood, urine, and microdialysis samples from subcutaneous adipose tissue beneath the dermal application site were collected for 24 h post-dosing. Total ^14^C-concentrations in plasma and microdialysates were quantified using accelerator mass spectrometry. The disposition of intravenously administered [^14^C]diclofenac was dose-linear. However, after topical administration, the fraction of total ^14^C absorbed (geometric mean and 95% confidence interval) was higher (*P* = .0019, 2-tailed, paired *t* test) for the microdose (0.48% and 0.34%–0.67%) compared with the therapeutic dose (0.13% and 0.07%–0.22%) (geometric mean ratio and 90% confidence interval: 3.79 and 2.41–5.98). Dose-normalized ^14^C-concentrations in microdialysates were low, variable, and did not differ between doses. Our study demonstrates the feasibility of quantifying ^14^C-concentrations in plasma and microdialysates following the topical administration of a microdose of [^14^C]diclofenac. The observed nonlinearity in systemic availability after topical dosing suggests that microdosing may not accurately predict the disposition of certain topical drugs at therapeutic doses.

**Significance Statement:**

We assessed whether a topically applied microdose of diclofenac could predict the systemic availability of a therapeutic dose. Results showed that systemic absorption was not dose-linear, indicating that microdosing may have limited use for predicting the pharmacokinetics of some topical drugs.

## Graphical abstract

**Figure:**
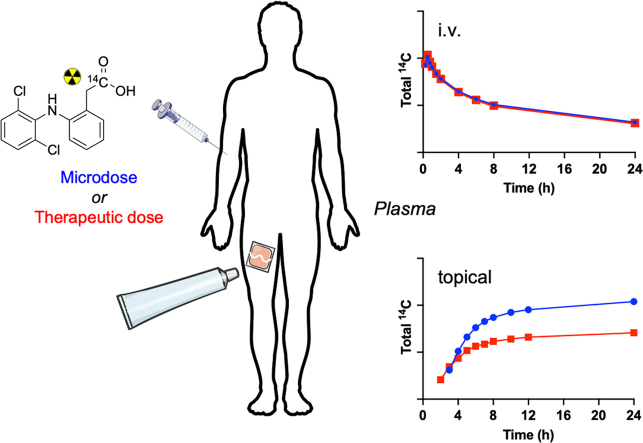

## 1 Introduction

Drugs are typically applied to the skin when the skin or underlying tissue layers are the targeted sites of action (Roberts et al, 2021). In these cases, systemic absorption is generally not desired, as this can cause systemic side effects. The primary barrier to dermal drug penetration is the stratum corneum, the outermost layer of the skin. Several factors influence the ability of topically administered drugs to penetrate the skin, including the physicochemical properties of the drug, the formulation used, and the condition of the skin. A key safety consideration for topical medications is whether and to what extent systemic exposure to the active ingredient occurs (Food and Drug Administration, 2019; European Medicines Agency, 2024). In pharmacokinetic (PK) studies of topical drugs, systemic exposure is evaluated to assess the rate and extent of absorption, distribution, and elimination from the body. Given the typically low systemic exposure following topical application, highly sensitive analytical methods are often required to characterize the PK profile. Dermal absorption studies are sometimes conducted using carbon-14 (^14^C)- or tritium (^3^H)-labeled drug substances, which can be quantified with high sensitivity and without prior knowledge of the metabolites (Wester and Maibach, 1992). In addition, microdialysis sampling is sometimes employed to measure unbound drug concentrations in the skin or underlying tissues (Holmgaard et al, 2010).

Microdosing, also known as phase 0 studies, involves the administration of a subtherapeutic dose of a drug (less than 1/100th of the pharmacologically active dose, less than 1/100th of the no-observed-adverse-effect level in animal toxicity studies, and less than 100 *μ*g for small-molecule drugs) to humans. These studies primarily aim to assess the drug’s PK and metabolism (Burt et al, 2020). A key prerequisite for the general applicability of microdosing is dose linearity, meaning that PK data obtained from microdoses should predict the PK data after therapeutic dosing (Lappin et al, 2006; Lappin et al, 2011). Regulatory authorities have recognized microdosing as a valuable tool in early-stage clinical drug development, providing crucial PK, including absorption, distribution, metabolism, and excretion data (ICH Expert Working Group, 2009). This allows for the early selection or rejection of drug candidates for further clinical development. Since microdosing involves very low doses, participants are exposed to minimal risk, and these studies require a reduced nonclinical safety testing package (ICH Expert Working Group, 2009). Due to the small doses involved, highly sensitive analytical techniques such as accelerator mass spectrometry (AMS) or liquid chromatography tandem mass spectrometry (LC-MS/MS) are essential (Keck et al, 2010; Burt et al, 2022).

In microdose studies, drug concentrations are typically assessed in plasma and excreta (ie, urine and feces), with limited data on tissue distribution. Positron emission tomography imaging using microdoses of radiolabeled drugs can measure tissue distribution, though this requires specialized research infrastructure and is limited by the short radioactive half-lives of the employed radionuclides (carbon-11, half-life: 20.4 min; fluorine-18, half-life: 109.8 min) (Wagner et al, 2011). We have recently proposed microdialysis (Müller, 2002) as a tool to measure unbound drug concentrations in target tissues such as skin, subcutaneous adipose tissue, or muscle tissue within the context of microdose studies (Oesterreicher et al, 2022). The feasibility of combining microdialysis with microdosing was demonstrated in healthy persons following intravenous administration of the fluoroquinolone antibiotic ciprofloxacin (Oesterreicher et al, 2022).

The aim of this study was to extend the microdosing concept to topically administered drugs. Specifically, we aimed to compare the systemic availability and plasma and tissue PK of the nonsteroidal anti-inflammatory drug diclofenac after topical administration of a ^14^C-microdose with that following the administration of a therapeutic dose. We conducted microdialysis sampling in subcutaneous adipose tissue of healthy volunteers beneath the dermal application site, along with blood and urine sampling.

## 2 Materials and methods

[^14^C]Diclofenac sodium (labeled in the carboxyl position, specific activity: 2.1 GBq/mmol and radiochemical purity: >99%, non–good-manufacturing practice grade) was purchased from Novandi Chemistry AB (Södertälje, Sweden) and supplied in glass vials containing either 740-kBq aliquots (for topical administration) or 7.4-kBq aliquots (for intravenous administration). Good-manufacturing practice-grade diclofenac sodium was obtained from the hospital pharmacy at the General Hospital Vienna (distributor: Gatt-Koller GmbH Pharmazeutika-Chemikalien, Absam, Austria; manufacturer: Henan Dongtain Pharm Co, Ltd). Voltaren 75-mg diclofenac sodium injection solution was obtained from Novartis Pharma GmbH. One ampoule of Voltaren contains 75 mg diclofenac sodium, 2 mg sodium pyrosulfite, 120 mg benzyl alcohol, 600 mg propylene glycol, mannitol, sodium hydroxide, and water in a total volume of 3 mL. Gel Cordes (Ichthyol-Gesellschaft Cordes, Hermanni & Co (GmbH & Co) KG) was obtained from the hospital pharmacy at the General Hospital Vienna. Gel Cordes is a hydrogel used for the extemporaneous preparation of topical drug formulations. It contains poloxamer 407, propylene glycol (20%), citric acid, di-sodium hydrogen phosphate dihydrate, and water and has a pH of 5.5–7.0. Human albumin CSL Behring 20% infusion solution (CSL Behring GmbH) was obtained from the hospital pharmacy at the General Hospital Vienna. Propylene glycol in United States Pharmacopoeia/European Pharmacopoeia quality was obtained from Merck KGaA. All other solvents and reagents were of analytical grade and were purchased from commercial suppliers.

### 2.1 Study drug formulation

#### 2.1.1 Topical administration

Individual doses were extemporaneously prepared within 24 h prior to administration. One aliquot of [^14^C]diclofenac sodium (740 kBq) was either dissolved in 0.6 mL propylene glycol (treatment period 1) or in 0.6 mL propylene glycol containing 120 mg diclofenac sodium (treatment period 3) and added dropwise to an aliquot of gel Cordes (6 g) under gentle stirring with a spatula. The gel was further stirred with the spatula until a homogenous mixture was obtained. From the homogenized gel, a weighted aliquot (approximately 100 mg, with exact weight recorded) was retained and stored at −80 °C for analysis of ^14^C-concentration with liquid scintillation counting (LSC).

#### 2.1.2 Intravenous administration

Individual doses were extemporaneously prepared within 24 h prior to administration. To a 100-mL physiological (0.9%, w/v) saline solution bag (freeflex + infusion bag, Fresenius Kabi), 0.5 mL of sterile 1 M sodium bicarbonate solution (Natriumbicarbonat “Fresenius” 1 molar Infusionszusatz Ampullen, Fresenius Kabi) was added. One aliquot of [^14^C]diclofenac sodium (7.4 kBq) was either dissolved in 0.6mL propylene glycol and 2.4 mL water for injection (treatment period 2) or in 3 mL of Voltaren 75-mg diclofenac sodium injection solution (treatment period 4) and passed through a Millex-GV sterile filter unit 0.22 *μ*m (Merck Millipore Ltd) into the infusion bag. The infusion bag was shaken to homogenize the solution, and a weighted aliquot of the infusion solution (approximately 1 g, with exact weight recorded) was removed and stored at −80 °C for analysis of ^14^C-concentration with LSC.

### 2.2 Study design

This was a prospective, single-center, 4-period, quadruple crossover, PK phase 1 study in healthy men and women conducted at the Medical University of Vienna from June to August 2021. The study was conducted in full accordance with the principles of the Declaration of Helsinki and its latest revision, the ICH harmonized tripartite guideline for Good Clinical Practice, the European Medicines Agency Note for Guidance on Good Clinical Practice, the Austrian Drug Law (Arzneimittelgesetz), and the Austrian Investigational Medical Devices Act (Medizinproduktegesetz). The trial was registered in the EudraCT database (2019-002842-19) and was approved by the Ethics Committee of the Medical University of Vienna and the Austrian Agency for Health and Food Safety. All participants gave oral and written informed consent before enrolment in the study.

After a screening visit, participants underwent 4 treatment periods in a fixed, nonrandomized order on 4 separate days with a washout period of at least 7 days between treatments. The washout period largely exceeded the terminal elimination half-life of diclofenac of 1–2 h. In treatment period 1, a topical microdose of [^14^C]diclofenac (nominal dose: 740 kBq) prepared as a homogenous mixture with 6 g of gel was applied on the intact, shaved skin of the thigh of each subject. In treatment period 2, each subject received a single intravenous microdose infusion of [^14^C]diclofenac (nominal dose: 7.4 kBq) diluted in physiological saline solution (100 mL) over 30 min. In treatment period 3, a combined topical dose of [^14^C]diclofenac (nominal dose: 740 kBq) and 120 mg diclofenac prepared as a homogenous mixture with 6 g of gel was applied on the intact, shaved skin of the thigh of each subject. In treatment period 4, each subject received a combined single-dose intravenous infusion of [^14^C]diclofenac (nominal dose: 7.4 kBq) and 75 mg diclofenac diluted in physiological saline solution (100 mL) over 30 min. For each treatment period, venous blood and urine sampling was conducted for 24 h after dosing. For treatment periods 1 and 3, subcutaneous microdialysis sampling beneath the dermal application site was additionally performed for 24 h after dosing.

No formal dosimetry calculation was performed for this study. Because of the high sensitivity of AMS, only very low [^14^C]diclofenac amounts were required for intravenous administration (nominal amount: 7.4 kBq). For the topical preparation, the administered amount of [^14^C]diclofenac was higher (nominal amount: 740 kBq). However, because the percentage of topically applied drug absorbed through the skin was expected to be low (∼6%) (Hui et al, 1998), the corresponding systemic exposure was expected to be ∼44 kBq. Because the energy of the β^−^ particles emitted in the decay of ^14^C is relatively low (156 keV), ^14^C-microdosing studies usually fall within risk category I (trivial risk, total effective dose < 0.1 mSv) according to the International Commission on Radiological Protection Publication 62 “Radiological Protection in Biomedical Research” (ICRP, 1992).

#### 2.3 Study population

The study enrolled healthy men and women aged 18–55 years, with a body mass index of 19–31 kg/m^2^, negative serology (human immunodeficiency virus, and hepatitis Bs-Ag and C-Ab) at screening and vital signs within the following ranges: systolic blood pressure 90–140 mm Hg, diastolic blood pressure 50–90 mm Hg, and pulse rate 50–90 bpm. Female participants with childbearing potential and at risk for pregnancy were allowed if they agreed to use effective methods of contraception. Participants had no clinically relevant disease, no evidence of anemia or hemolysis at screening, no contraindication against administration of diclofenac, or any other nonsteroidal anti-inflammatory drug; had not taken other medications within the last 7 days (exception: hormonal contraceptives for female participants); had no allergy to any of the active or inactive ingredients of the study medication; did not smoke >5 cigarettes per day; had no history of drug or alcohol abuse; did not participate in a clinical trial within 30 days before the start of the study; and had not donated blood in excess of 500 mL within a period of 4 weeks prior to study start. Paracetamol was allowed as concomitant medication as needed until 48 h prior to study medication intake.

### 2.4 Sample collection

#### 2.4.1 Microdialysis sampling

After topical [^14^C]diclofenac administration (treatment periods 1 and 3), total ^14^C-concentrations were sampled from subcutaneous adipose tissue beneath the dermal application site using microdialysis. The microdialysis method allows sampling of the unbound fraction of an analyte from interstitial space fluid by insertion of a microdialysis catheter into the tissue of interest (Müller, 2002). The microdialysis catheter is equipped with a semipermeable membrane at its tip and is constantly perfused at a predefined flow rate with physiological solution.

Approximately 24 h before topical drug administration, the lateral front of the thighs of each subject was marked with a precise area of 10 × 15 cm = 150 cm^2^ as the site of application using a skin marker. The marked area was shaved using a hypoallergic shaving gel and washed with mild soap and water, and patted dry. In the morning of the study day, two 63 Microdialysis Catheters (M Dialysis AB) with a membrane length of 10 mm and a molecular mass cutoff of 20 kDa were aseptically inserted into subcutaneous adipose tissue below the marked application area. To ensure that the microdialysis probes were properly placed below the application area and to measure the depth of probe insertion, an ultrasound scan was performed. The gel containing solely [^14^C]diclofenac (treatment period 1) or a mixture of [^14^C]diclofenac and unlabeled diclofenac (treatment period 3) was applied on the shaved skin area, which was then covered with a transparent film dressing (3M Tegaderm). Microdialysis probes (MD1 and MD2) were perfused with phosphate-buffered saline solution containing 1% (w/v) human serum albumin (HSA) at a flow rate of 2 *μ*L/min by means of a portable battery-driven microdialysis pump (107 Microdialysis Pump, M Dialysis AB). After collection of 1 predose sample, microdialysis samples were continuously collected in 1-h time intervals until 12 h after topical drug administration, then at 22–23, and 23–24 h after drug administration.

Because of a lack of equilibrium between interstitial space fluid and the perfusion medium, concentrations in microdialysates are usually corrected by a factor that is determined by a probe calibration procedure called retrodialysis to obtain true tissue concentrations. The underlying assumption is that the exchange process across the semipermeable membrane of the microdialysis probe is equal in both directions. Therefore, a perfusion solution containing a known concentration of the analyte is pumped through the microdialysis system. By measuring the analyte concentration in the dialysate fluid, the relative loss of analyte across the semipermeable membrane, which is assumed to equal the relative recovery, can be calculated as 100 − (concentration_dialysate_/concentration_perfusate_ × 100) (Plock and Kloft, 2005).

Retrodialysis experiments were performed at the end of the 24-h sampling phase for both treatment periods. For retrodialysis, the catheter was perfused with phosphate-buffered saline solution containing either [^14^C]diclofenac (0.112 ng/mL) (treatment period 1) or [^14^C]diclofenac and unlabeled diclofenac (100 ng/mL) (treatment period 3) at a flow rate of 2 *μ*L/min. After 60 min of equilibration, microdialysate samples were collected from 1 to 1.5 h and from 1.5 to 2 h after start of retrodialysis. To study the influence of HSA on the calculated loss values, the perfusate of 1 of the 2 microdialysis probes contained 1% (w/v) HSA, while the other did not. At the end of the experiment, microdialysis catheters were removed. Microdialysis samples were stored at –80 °C until determination of total ^14^C-concentrations using AMS.

#### 2.4.2 Blood and urine sampling

Venous blood samples (9 mL) were collected via an indwelling catheter into ethylenediaminetetraacetic acid tubes at predose, 1, 2, 3, 4, 5, 6, 7, 8, 10, 12, and 24 h after topical drug administration (treatment periods 1 and 3) and at predose, 0.25, 0.5, 0.75, 1, 1.5, 2, 4, 6, 8, and 24 h after intravenous drug administration (treatment periods 2 and 4). Blood samples were centrifuged (2600 × g, 10 min, 4 °C) to obtain plasma, and plasma aliquots were stored at –80 °C until determination of total ^14^C-concentrations with AMS (all treatment periods) and diclofenac concentrations with LC-MS/MS (only for treatment periods 3 and 4). Urine was collected for all 4 treatment periods at predose and at 0–6, 6–12, and 12–24 h after drug administration. The urine volume was recorded, and urine aliquots were stored at –80 °C until determination of total ^14^C-concentrations with LSC.

### 2.5 Sample analysis

Sample analysis was performed at the Korea Institute of Radiological and Medical Sciences.

#### 2.5.1 AMS analysis

Analysis of total ^14^C in plasma and microdialysate samples was performed using AMS (0.5-keV National Electrostatics Corporation Pelletron). Sample pretreatment, which converts the carbon within the samples to graphite via a 2-step process of oxidation and reduction, was performed using the procedure described by Ognibene et al (2003) with minor modifications. For microdialysates, 1 mL of tributyrin (carrier carbon) was added to the samples to bring the total carbon content to the optimal amount for efficient conversion to graphite. This addition also ensures that >99% of the carbon in the sample was derived from the carbon carrier, which simplifies the total drug-related concentration calculations. Plasma was prepared without tributyrin addition, using the native carbon (4.2% average carbon content) as the carbon source to calculate the total drug-related concentration (Vogel et al, 2011). All samples were dried under vacuum centrifugation and subsequently oxidized to carbon dioxide by heating at 900 °C for 3 h in the presence of copper oxide as an oxygen source. The carbon dioxide was then cryogenically transferred to a reduction vial under vacuum and reduced to graphite in the presence of zinc dust and iron powder at 525 °C for 3 h. Graphitized samples were pressed into aluminum targets and analyzed for ^14^C:^12^C ratios with AMS. Oxalic Acid II standards (SRM 4990C, NIST) and IAEA-certified materials (C3 and C8) were run with each batch for isotope ratio normalization and as quality controls. To calculate concentrations (in nanogram diclofenac equivalents per milliliter, ng-eq/mL), the following equation was applied:

$$\text{Concentration}\;\left(\text{ng}‐\text{eq}/\text{mL}\right)=\frac{\left(R_{\text{meas}}\;-\;R_{\text{bkg}}\right)\;\left(\frac{0.01356\;\text{dpm}}{\text{mgC}\;}\right)\;\left(C_{\text{conc}}\right)\;}{\left(\text{SpecAct}\;\text{dpm}/\text{ng}\right)}$$

where *R*_meas_ is the measured ^14^C:^12^C ratio in the sample (in units of modern), *R*_bkg_ is the background ^14^C:^12^C ratio (determined from the predose samples), C_conc_ is the mass of carbon per unit volume of the sample (in mg/mL), 0.01356 is the conversion factor from modern units to dpm/mg carbon, and SpecAct is the specific activity of [^14^C]diclofenac (in dpm/ng). The specific activity of [^14^C]diclofenac used in the study was 397 dpm/ng (2.1 GBq/mmol). The AMS quantification method achieved a lower limit of quantification (LLOQ) of 0.029 pg/mL for the analysis of microdialysate samples.

#### 2.5.2 Liquid scintillation counting

LSC was used to measure total ^14^C in urine samples as well as in the aliquots of the administered drug formulations. Analyses were performed using a Tri-carb 5110 TR (PerkinElmer). To 2-mL aliquots of urine, 18 mL Ultima Gold LSC cocktail was added, the vial capped, mixed by vortex action, and allowed to rest in the instrument overnight for dark adaptation prior to LSC. Gel samples were dissolved in 1 mL of 80% ethanol. Aliquots of the dissolved gel samples (80 mg) or aliquots of the intravenous infusion solution (100 *μ*L) were mixed with 16 mL LSC cocktail and analyzed by LSC. A set of factory-provided external standards were employed to correct for counting efficiency and quenching effects (quench curve). Count times were set to achieve <5% imprecision. The radioactivity in urine samples was expressed in dpm/mL and then converted to concentrations of diclofenac and its metabolites (ng-eq/mL) using the specific activity of [^14^C]diclofenac (397 dpm/ng). The LLOQ for LSC was 1.5 dpm/mL. Radioactivity in aliquots of the drug formulations was converted into kBq values.

#### 2.5.3 LC-MS/MS analysis

##### 2.5.3.1 Chemicals and reagents

Formic acid and diclofenac sodium salt were purchased from Sigma-Aldrich. Diclofenac-D_4_ (used as an internal standard of diclofenac) was purchased from Toronto Research Chemicals. High-pressure liquid chromatography-grade acetonitrile and water were purchased from J.T. Baker.

##### 2.5.3.2 Instrumentation

The LC-MS/MS system was composed of a Waters UPLC system with a Xevo G2-XS mass spectrometer equipped with an electrospray ionization source. Chromatographic separation was achieved with an HSS T3 column (100 × 2.1 mm, 1.8 *μ*m; Waters) connected to a Security Guard Pre-Column (C18, 4 × 2 mm; Phenomenex). The column oven temperature was maintained at 40 °C. The mobile phase consisted of 0.1% (v/v) formic acid in distilled water (A) and 0.1% (v/v) formic acid in acetonitrile (B). A gradient program was used for the chromatographic separation, with a flow rate of 0.4 mL/min as follows: %B: 5%: 0–2.5 min, 5%–95%: 2.5–3.5 min, 95%–5%: 3.5–3.6 min, and 5%: 3.6–6.0 min. The injection volume was 3 *μ*L. Drying gas (nitrogen) temperature was set at 250 °C and capillary voltage at 1.5 kV. Multiple-reaction monitoring detection was used, with argon as the collision gas. The precursor-product ion pairs monitored were m/z 296.07 → 215.02 for diclofenac and m/z 300.1 → 219.1 for diclofenac-D_4_ (internal standard).

##### 2.5.3.3 Preparation of calibration standards

A stock solution of diclofenac sodium (1 mg/mL as diclofenac) was prepared in distilled water. Diclofenac-D_4_ was dissolved in 10% methanol. The stock solution was stored at −20 °C and brought to room temperature before use. Working standard solutions of diclofenac were prepared in 10% methanol with final concentrations ranging from 1 to 100 *μ*g/mL. Calibration standards were prepared by spiking 99 *μ*L of blank human plasma with 1 *μ*L of the appropriate working standard solution. This yielded calibration standards at concentrations of 10, 20, 50, 100, 300, 500, and 1000 ng/mL. An internal standard solution was prepared by diluting the diclofenac-D_4_ stock solution in acetonitrile to a final concentration of 100 ng/mL. The LLOQ was set to the lowest calibration standard concentration (10 ng/mL).

##### 2.5.3.4 Sample preparation

A 300-*μ*L aliquot of acetonitrile containing 100 ng/mL of diclofenac-D_4_ (internal standard) was added to a 100-μL plasma sample. After mixing, the sample was centrifuged (14,000 × g, 10 min, 4 °C). The supernatant was then collected and transferred to a vial for LC-MS/MS analysis.

### 2.6 Safety

Adverse events were closely monitored throughout the study. All participants underwent a screening examination at the beginning and an end-of-study examination, which included a physical exam, vital sign measurements, an electrocardiogram, and blood and urine samples for laboratory tests. These tests included hematology, clinical chemistry, coagulation, virology, urinalysis, urine drug screening, and a pregnancy test for female participants.

### 2.7 PK and statistical analysis

Due to the exploratory nature of the study, no formal sample size calculation was conducted. A sample size of 8 was selected. Plasma diclofenac (parent drug, measured with LC-MS/MS) and plasma and tissue total ^14^C (comprising parent [^14^C]diclofenac and its radiolabeled metabolites, measured with AMS) concentration-time curves were analyzed using noncompartmental analysis with Phoenix WinNonlin version 8.5.2.4 (Certara). For treatment period 4 (administration of the intravenous therapeutic dose), diclofenac plasma PK was characterized in terms of maximum plasma concentration, time to maximum plasma concentration, area under the concentration-time curve from 0 to 24 h (AUC_0–24_), area under the concentration-time curve from 0 to infinity (AUC_0-inf_), total clearance from plasma, volume of distribution, volume of distribution at steady state, and mean residence time. To facilitate comparison of PK parameters following the microdose versus the therapeutic dose, total ^14^C-concentrations in plasma, tissue, and urine were normalized to the individual administered dose of diclofenac (in *μ*g) and expressed as ng-eq/mL diclofenac per *μ*g administered diclofenac. AUC_0–24_ was calculated based on the dose-normalized concentration-time profiles of total ^14^C. For tissue concentration-time profiles measured with microdialysis, sampling time points at the end of each sampling interval were assumed. The fraction of total ^14^C absorbed after topical administration (F_abs_) was calculated either as (AUC_0–24,topical_/AUC_0–24,i.v_) × 100 or as (% dose excreted in urine [topical]/% dose excreted in urine [i.v.]) × 100. The rate of increase of plasma concentrations following topical administration was determined by linear regression analysis of the linear part of the natural log-transformed plasma concentration-time curves (ie, from 3 to 8 h after dosing) using Microsoft Excel 2019 MSO. Data are presented as geometric mean with 95% confidence intervals unless stated otherwise. For geometric mean ratios (GMRs), a 90% confidence interval is stated. For comparison of PK parameters between the microdose and the therapeutic dose, a 2-tailed, paired *t* test was applied to the natural log-transformed data. Prior to analysis, the normality of the paired differences between log-transformed values was assessed using the Shapiro–Wilk test. The level of statistical significance was set to a *P* value of < .05. Statistical testing was performed using Microsoft Excel.

## 3 Results

### 3.1 Demographic characteristics

Seven men and 3 women were screened, of whom 6 men and 2 women were enrolled and completed the study. Their mean (±SD) age was 32 ± 9 years (range, 23–51 years) with a mean (±SD) body mass index of 23.9 ± 2.3 kg/m^2^ (range, 21.5–28.2 kg/m^2^).

### 3.2 Administered doses

The mean (±SD) administered topical doses of [^14^C]diclofenac sodium were 412 ± 37 kBq in treatment period 1 and 341 ± 62 kBq in treatment period 3, corresponding to 62 ± 6 *μ*g and 51 ± 9 *μ*g of unlabeled diclofenac sodium, respectively. In treatment period 3, 120 mg of unlabeled diclofenac sodium was coadministered with the radiotracer. The mean (±SD) administered intravenous doses of [^14^C]diclofenac sodium were 6.3 ± 0.2 kBq in treatment period 2 and 6.6 ± 0.3 kBq in treatment period 4, corresponding to 0.95 ± 0.03 *μ*g and 1.0 ± 0.04 *μ*g of unlabeled diclofenac sodium, respectively. In treatment period 4, 75 mg of unlabeled diclofenac sodium was coadministered with the radiotracer.

### 3.3 Plasma PKs

**Fig. 1:**
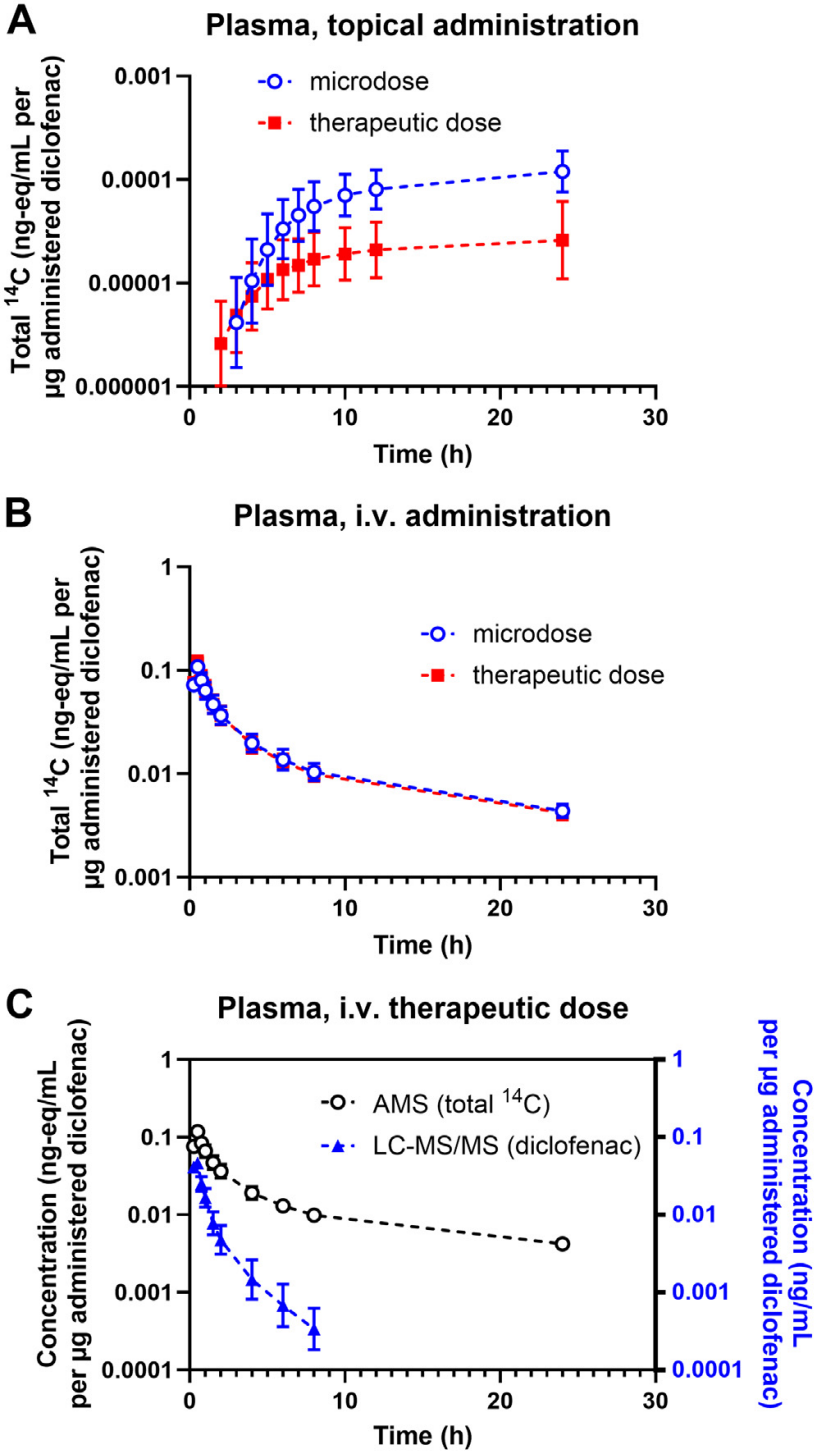
Geometric mean (95% confidence interval, *n* = 8) dose-normalized plasma concentration-time curves of total ^14^C (ng-eq/mL diclofenac per *μ*g administered diclofenac) after topical (A) or intravenous (B) administration of a microdose or therapeutic dose of diclofenac. (C) Geometric mean (95% CI, *n* = 8) dose-normalized plasma concentration-time curves of total ^14^C (left y-axis: ng-eq/mL diclofenac per *μ*g administered diclofenac, measured with AMS) or of parent diclofenac (right *y*-axis, ng/mL per *μ*g administered diclofenac, measured with LC-MS/MS) after intravenous administration of a therapeutic dose of diclofenac. The deviation between the AMS and the LC-MS/MS data is attributed to [^14^C]metabolite exposure not detectable by LC-MS/MS. CI, confidence interval.

Dose-normalized plasma concentration-time curves of total ^14^C (including parent [^14^C]diclofenac and its ^14^C-labeled metabolites) following topical administration of either a microdose or a therapeutic dose of diclofenac are shown in Fig. 1A. In both periods, plasma concentrations began to rise approximately 2 h after administration and appeared to plateau by the end of the sampling period. Plasma concentrations increased at a higher rate (*P* = .0001) for the microdose compared with the therapeutic dose (microdose: 0.49 h^−1^, 0.42–0.58 h^−1^; therapeutic dose: 0.23 h^−1^, 0.19–0.29 h^−1^; GMR: 2.1, 1.8–2.5). Dose-normalized plasma AUC_0–24_ was higher (*P* = .0021) for the topical microdose compared with the topical therapeutic dose, with a GMR of 3.84 (2.40–6.15; Table 1). In contrast, the dose-normalized ^14^C-plasma concentration-time curves following intravenous administration of a microdose or therapeutic dose of diclofenac were nearly identical (Fig. 1B), with a GMR of dose-normalized plasma AUC_0–24_ between the intravenous microdose and therapeutic dose of 1.01 (0.96–1.07; *P* = .72; Table 1). Given the higher dose-normalized plasma exposure to total ^14^C following topical administration of the microdose compared to the therapeutic dose (Fig. 1A), the fraction of total ^14^C absorbed (F_abs_) was higher (*P* = .0019) for the microdose than for the therapeutic dose, with a GMR of 3.79 (2.41–5.98; Table 1).

**Table 1:** Summary of PK parameters for the 4 treatment periods (based on quantification of total ^14^C with AMS or LSC)

Parameter	Topical Therapeutic Dose	Topical Microdose	Intravenous Therapeutic Dose	Intravenous Microdose
Plasma AUC_0–24_ (ng-eq·h/mL)	0.00043 (0.00024–0.00077)	0.00165 (0.00114–0.00239)^a^	0.342 (0.302–0.387)	0.346 (0.307–0.390)
GMR		3.84 (2.40–6.15)		1.01 (0.96–1.07)
F_abs_ (%)^b^	0.13 (0.07–0.22)	0.48 (0.34–0.67)^a^	-	-
GMR		3.79 (2.41–5.98)		-
Tissue AUC_0–24_ (ng-eq·h/mL) MD1^c^	0.000053 (0.000019–0.000147)	0.000110 (0.000027–0.000445)	-	-
GMR		2.07 (0.43–10.0)		-
Tissue AUC_0–24_ (ng-eq·h/mL) MD2^c^	0.000043 (0.000010–0.000183)	0.000062 (0.000012–0.000333)		
GMR		1.44 (0.37–5.61)		
Excreted in urine (% administered dose)^d^	0.08 (0.04–0.16)	0.30 (0.22–0.42)^a^	42.3 (33.8–53.0)	45.5 (38.2–54.2)
GMR		3.61 (2.40–5.44)		1.08 (0.92–1.26)
F_abs_ (%)^e^	0.20 (0.11–0.37)	0.67 (0.50–0.89)^a^		
GMR		3.36 (2.09–5.38)		

All values are given as geometric mean (95% CI; *n* = 8 except for tissue AUC_0–24_ MD1 with *n* = 7). For the geometric mean ratio (GMR) between microdose and therapeutic dose, the 90% CI is stated. All parameters are based on quantification of total ^14^C (nanogram diclofenac equivalents, ng-eq) with AMS (plasma, microdialysates) or LSC (urine). All concentrations were normalized to the individually administered doses of diclofenac (in *μ*g). Values for microdialysates are not recovery-corrected. AUC_0–24_, area under the concentration-time curve from 0 to 24 h; F_abs_, fraction absorbed (%) after topical administration. CI, confidence interval.

a *P* < .01 versus microdose (2-tailed, paired *t* test).

b Calculated from plasma data.

c 2 microdialysis probes (MD1 and MD2) were inserted in each subject and tissue AUC_0–24_ values are reported for both probes separately.

d Cumulative urinary excretion from 0 to 24 h after dosing.

e Calculated from urinary excretion data.

Apart from total ^14^C-concentrations, plasma concentrations of parent diclofenac following topical or intravenous administration of the therapeutic dose were analyzed using LC-MS/MS. While diclofenac plasma concentrations were below the LLOQ of 10 ng/mL after topical administration, they were quantifiable after intravenous administration. Dose-normalized diclofenac plasma concentration-time profiles following intravenous administration of the therapeutic dose were compared with AMS-measured total ^14^C-concentrations (Fig. 1C). The profiles diverged, indicating substantial metabolism of diclofenac during the sampling period. The geometric mean percentage of AUC_0–24_ of parent diclofenac in plasma relative to total ^14^C was 14.0% (12.5%–15.7%). Plasma PK data for parent diclofenac following intravenous administration of the therapeutic dose are presented in Table 2.

**Table 2:** Summary of PK parameters after intravenous administration of a therapeutic dose (75 mg) of diclofenac (based on quantification of diclofenac with LC-MS/MS)

Parameter	
C_max_ (ng/mL)	3456 (3100–3852)
t_max_ (h)	0.5
AUC_0–24_ (ng·h/mL)	3598 (2946–4396)
AUC_0–inf_ (ng·h/mL)	3672 (2982–4521)
CL (L/h)	20.4 (16.6–25.1)
V_z_ (L)	52.9 (45.9–60.9)
V_ss_ (L)	24.1 (22.1–26.1)
MRT (h)	1.18 (0.95–1.47)

All values are given as geometric mean (95% CI; *n* = 8). All parameters are based on quantification of diclofenac in plasma with LC-MS/MS.

CI, confidence interval; C_max_, maximum plasma concentration; t_max_, time to maximum plasma concentration; AUC_0–24_, area under the concentration-time curve from 0 to 24 h; AUC_0–inf_, area under the concentration-time curve from 0 to infinity; CL, total clearance from plasma; V_z_, volume of distribution; V_ss_, volume of distribution at steady state; MRT, mean residence time.

### 3.4 Tissue PKs

**Fig. 2:**
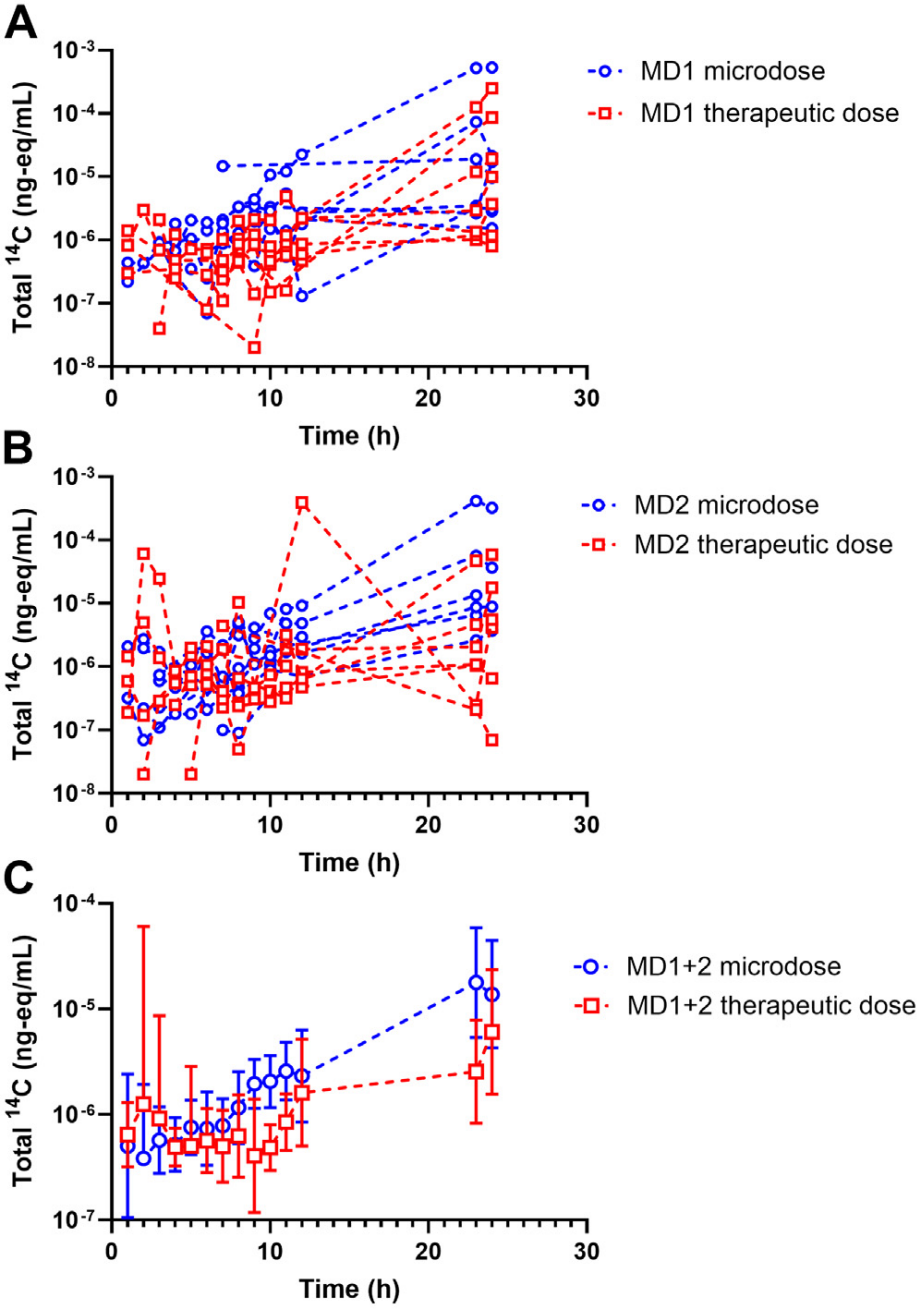
Individual dose-normalized concentration-time curves of total ^14^C (ng-eq/mL diclofenac per *μ*g administered diclofenac) in microdialysates collected from subcutaneous adipose tissue after topical administration of a microdose or therapeutic dose of diclofenac. In each subject, 2 microdialysis probes were inserted (A: MD1, *n* = 7, and B: MD2, *n* = 8). The shown concentrations are not corrected for the recovery of the microdialysis probes. In C, geometric mean (95% CI) concentration-time curves across both microdialysis probes are shown for the microdose and therapeutic dose. CI, confidence interval.

Two microdialysis probes (MD1 and MD2) were inserted into the subcutaneous adipose tissue beneath the topical drug administration site to measure the tissue PK of total ^14^C using AMS. The mean (±SD) depth of the probe tip was 8.3 ± 3.5 mm (MD1) and 8.4 ± 4.4 mm (MD2) for the topical microdose, and 10.7 ± 4.3 mm (MD1) and 10.8 ± 3.8 mm (MD2) for the topical therapeutic dose. For both MD1 and MD2, some data points were missing in certain participants due to limitations in sample volume, improper sealing of sample vials, or issues during the sample preparation process (Table 3). For probe calibration using the retrodialysis method, the perfusion solution contained 1% HSA for MD2, but no HSA was included for MD1. The mean (±SD) percentage loss of diclofenac during retrodialysis was 84% ± 9% for the microdose and 87% ± 6% for the therapeutic dose in MD1. In contrast to MD1, the loss could not be determined for MD2 because microdialysate concentrations were generally higher than perfusate concentrations in most cases. Due to this inconsistency in probe calibration, we only report the nonrecovery-corrected tissue concentration-time profiles of total ^14^C (Fig. 2). ^14^C-concentrations in microdialysates were low and highly variable for both probes and across both treatment periods. In most participants, the concentration-time curves gradually increased over time, with the highest concentrations observed at the end of the sampling period. For both microdialysis probes, dose-normalized AUC_0–24_ values were not different between the microdose and therapeutic dose (MD1: *P* = .47, GMR: 2.07, 0.43–10.0; MD2: *P* = .67, GMR: 1.44, 0.37–5.61, Table 1).

**Table 3:** Missing data points from microdialysis sampling

Participant	MD1		MD2	
	Microdose	Therapeutic Dose	Microdose	Therapeutic Dose
1	-	-	1, 5, 9, 12, 24 h	10, 24 h
2	-	-	6, 12, 23, 24 h	-
3	8 h	-	predose, 12 h	3 h
4	-	2 h	4, 10, 23, 24 h	-
5	-	-	1, 2, 5 h	9 h
6	-	9 h	predose	-
7	all	all	-	7, 8, 9, 12, 23, 24 h
8	-	-	-	-

Stated are the sampling time points (h) of each missing sample for microdialysis probes 1 (MD1) and 2 (MD2).

### 3.5 Urinary excretion data

**Fig. 3:**
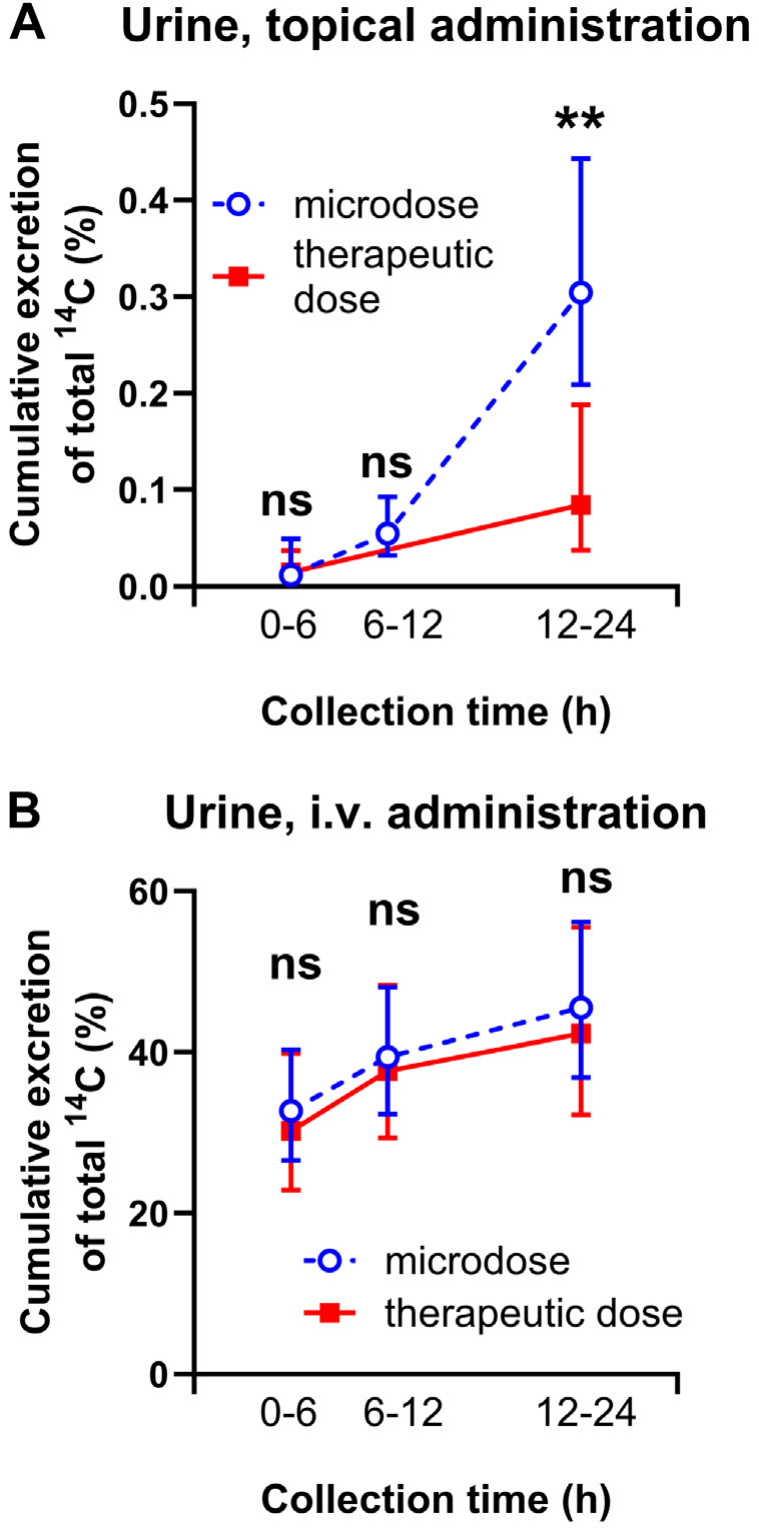
Geometric mean (95% CI, *n* = 8) cumulative urinary excretion of total ^14^C (% of administered [^14^C]diclofenac dose) following topical (A) or intravenous (B) administration of a microdose or therapeutic dose of diclofenac. ∗∗, *P* < .01, 2-tailed, paired *t* test. CI, confidence interval.

Cumulative urinary excretion of total ^14^C following topical or intravenous administration is shown in Figs. 3A and 3B, respectively. In line with the higher dose-normalized plasma exposure observed after topical administration of the microdose (Fig. 1A), cumulative urinary ^14^C-excretion from 0 to 24 h post-dosing was higher (*P* = .0013) for the microdose compared with the therapeutic dose, with a GMR of 3.61 (2.40–5.44, Table 1; Fig. 3A). In contrast, no difference (*P* = .46) in cumulative urinary ^14^C-excretion was observed between the microdose and therapeutic dose following intravenous administration (Table 1; Fig. 3B). Consistent with F_abs_ derived from plasma data, F_abs_ calculated from urinary excretion data was also higher (*P* = .0038) for the microdose than for the therapeutic dose, with a GMR of 3.36 (2.09–5.38; Table 1).

### 3.6 Safety

No serious adverse events were reported during the study. One participant experienced a mild headache, whereas another reported a mild burning sensation at the site of the intravenous diclofenac injection. Both adverse events were self-resolving without the need for treatment and were considered possibly or probably related to the study drug. Throughout the study, there were no clinically significant changes observed in clinical laboratory assessments, vital signs, electrocardiogram readings, or physical examination results.

## 4 Discussion

The aim of this study was to evaluate whether a topically administered microdose of a drug could predict the systemic availability, plasma, and tissue PK of a topical therapeutic dose. To address this, we used diclofenac, a nonsteroidal anti-inflammatory drug commonly applied topically for the relief of pain and inflammation in muscles and joints, as a model compound (Goh and Lane, 2014). Unlike many other topically administered drugs, which act on superficial skin layers, diclofenac targets deeper dermal, subcutaneous, and muscle tissues (Dancik et al, 2012). We compared the systemic availability and PK of a topically administered, extemporaneously prepared formulation containing either a microdose (∼60 *μ*g) of [^14^C]diclofenac alone or [^14^C]diclofenac mixed with a therapeutic dose (120 mg), covering an approximately 2000-fold dose range, in healthy volunteers. The topical therapeutic dose of diclofenac was chosen based on the recommended daily dose for a marketed diclofenac gel (Voltaren Emulgel 1.0%) (Voltaren). Blood and urine samples were collected over 24 h following topical dosing, and microdialysis was performed to assess subcutaneous adipose tissue PK. In addition, participants underwent treatment periods after intravenous infusion of a microdose or therapeutic dose of diclofenac, which allowed us to calculate F_abs_ after topical administration, based on plasma exposure or urinary excretion data.

Since the sensitivity of LC-MS/MS was insufficient to quantify parent diclofenac concentrations in plasma and microdialysates after topical administration, we used AMS as a more sensitive analytical technique. Only a few studies have employed AMS to assess topical absorption (Gilman et al, 1998; Iyer et al, 2012). Due to the technical challenges of chromatographically fractionating low-volume microdialysates, we quantified total ^14^C-concentrations in microdialysates and plasma rather than parent [^14^C]diclofenac. Because diclofenac undergoes significant metabolism (Stierlin and Faigle, 1979), ^14^C-concentrations in plasma and urine included a substantial proportion of ^14^C-labeled metabolites (Fig. 1C). It seems unlikely that diclofenac metabolism and excretion are dose-dependent because dose-normalized total ^14^C-concentration-time profiles in plasma (Fig. 1B) and urinary excretion data (Fig. 3B) following intravenous administration of either a microdose or a therapeutic dose were similar (Table 1). The observed dose linearity in the PK of intravenously administered [^14^C]diclofenac aligns with existing literature on intravenous small-molecule microdosing (Lappin et al, 2011; van Nuland et al, 2019). It is, therefore, unlikely that considering parent [^14^C]diclofenac, rather than total ^14^C, would have significantly altered the main findings of our study. Previous clinical microdialysis studies have reported very low and variable diclofenac concentrations in microdialysates, often below the limit of quantification of the employed assays (Jorda et al, 2022). In contrast, we were able to quantify ^14^C-concentrations in microdialysates with AMS due to its high sensitivity.

An important finding of our study was the higher rate and extent of systemic absorption of total ^14^C for the topical microdose compared with the therapeutic dose. A literature review of 138 dermal absorption experiments with 98 different substances found that in 63% of the studies, relative dermal absorption decreased at higher dermal doses (Buist et al, 2009). This nonlinearity in dermal absorption was especially evident for poorly water-soluble compounds and may be explained by saturation of the skin’s absorption capacity at higher dermal doses (Buist et al, 2009; Kissel, 2011). One mechanism that may potentially play a role in the case of diclofenac is saturation of membrane transporter activity in the skin at high diclofenac concentrations. The adenosine triphosphate-binding cassette transporters P-glycoprotein/adenosine triphosphate-binding cassette B1 and breast cancer resistance protein/adenosine triphosphate-binding cassette G2 are expressed in the skin and may mediate the influx of their substrates from the epidermis into the dermis (Hashimoto et al, 2013). While data on transporter interactions of diclofenac are limited, one study suggests that it is transported by human breast cancer resistance protein, but not by P-glycoprotein (Lagas et al, 2009). Brunner et al. (Brunner et al, 2011) measured plasma and tissue PK of diclofenac after repeated administration of an investigational topical diclofenac formulation at 2 dose levels (1% and 2.5%) and found a dose-normalized plasma AUC approximately 1.4-fold higher for the lower dose level (4754 vs 3368 pg·h/mL). This is consistent with our finding of a higher F_abs_ for the microdose compared with the therapeutic dose (Table 1). However, it should be noted that the dose range in the study by Brunner et al. (daily doses: 5 and 12.5 mg over 3 days) was not comparable to the dose range in our study.

F_abs_ for total ^14^C following topical administration of [^14^C]diclofenac in our study ranged from 0.1% to 0.7%, which was approximately 10–70 times lower than 6.6% F_abs_ reported in a previous study by Hui et al (1998), based on urinary excretion data. One possible explanation for this discrepancy is that our extemporaneously prepared diclofenac formulation was not optimized for dermal absorption, whereas Hui et al. (1998) added [^14^C]diclofenac to a marketed diclofenac lotion (Pennsaid topical solution™) containing 48.1% DMSO as an absorption enhancer. In addition, the shorter sampling period in our study (24 h vs 120 h) may also contribute to the observed differences in F_abs_ between the 2 studies. It cannot be excluded that the use of a formulation optimized for dermal absorption might have reduced the degree of nonlinearity in diclofenac absorption observed in this study.

Before conducting this study, the feasibility of using microdialysis and AMS to quantify [^14^C]diclofenac from microdoses or therapeutic doses was assessed in vitro (Langer et al, 2022). In this setup, microdialysis was performed in an immersion solution simulating interstitial space fluid, with defined diclofenac concentrations in the presence of varying HSA concentrations. The in vitro study demonstrated dose linearity over a 1000-fold dose range for diclofenac diffusion across the microdialysis membrane. However, in the in vitro study, a considerable discrepancy was observed between the relative loss values obtained through retrodialysis and the relative recovery values, irrespective of the employed dose, which resulted in an underestimation of the true unbound diclofenac concentrations in the immersion solution when applying correction factors derived from retrodialysis (Langer et al, 2022). This effect became more pronounced as the concentration of HSA in the immersion solution increased, leading to the conclusion that determining true tissue concentrations using microdialysis is particularly challenging for highly protein-bound drugs like diclofenac (which has a plasma protein binding of 99.8%) (Borgå and Borgå, 1997; Jorda et al, 2022; Langer et al, 2022). In general, the perfusion solution for retrodialysis should have the same composition as the perfusion solution for forward microdialysis. In our study, the perfusion solution for microdialysis contained 1% HSA to reduce nonspecific binding and improve recovery for highly protein-bound drugs (Hammarlund-Udenaes, 2017). However, during retrodialysis, when HSA was included in the perfusion solution, loss values could not be determined because some participants exhibited higher ^14^C-concentrations in the microdialysates than in the perfusion solution. A similar observation was made by Jorda et al (2022) in their in vitro microdialysis study with diclofenac. In contrast, retrodialysis without HSA in the perfusion solution resulted in loss values around 85%, which are unlikely to represent the actual diclofenac recovery, because these values were approximately 5% in the in vitro setup (Langer et al, 2022). As a result of these uncertainties in probe calibration, we analyzed only nonrecovery-corrected ^14^C-microdialysate concentrations and focused on the relative comparison of the tissue concentration-time profiles between the microdose and therapeutic dose.

Penetration of topically administered diclofenac into deeper dermal tissues is believed to occur through both direct diffusion from the application site and redistribution of systemically absorbed diclofenac (Dancik et al, 2012). An in vitro study found that during absorption of diclofenac through viable human skin, diclofenac is not metabolized (Tanojo et al, 1999). Despite higher dose-normalized ^14^C-plasma concentrations for the topical microdose compared with the therapeutic dose (Fig. 1A), dose-normalized ^14^C-microdialysate concentrations did not differ (Fig. 2; Table 1). This may be attributed to high interindividual variability in microdialysate concentrations. For the first 8 h after topical dosing, microdialysate ^14^C-concentrations remained near background levels, gradually increasing toward the end of the sampling period (Fig. 2). This pattern mirrored that observed in the plasma concentration-time curves (Fig. 1A), which continuously rose over the sampling period. The lag times for ^14^C-concentrations to appear in plasma (Fig. 1A) or microdialysates (Fig. 2) following topical administration were longer than those reported in previous microdialysis studies with marketed diclofenac formulations optimized for dermal delivery (Müller et al, 1997; Brunner et al, 2005; Brunner et al, 2011), suggesting that our formulation had a lower dermal absorption rate. Due to the inability to correct microdialysate concentrations for probe recovery to obtain true unbound tissue concentrations, and because we quantified total ^14^C rather than parent [^14^C]diclofenac, our microdialysate concentrations cannot be directly compared with previously published diclofenac tissue concentration-time profiles (Müller et al, 1997; Brunner et al, 2005; Brunner et al, 2011). As a result, no conclusions can be drawn regarding whether diclofenac tissue concentrations were within a pharmacologically effective range. However, since we used an extemporaneously prepared diclofenac formulation rather than a marketed topical product, and given that the primary objective of our study was to assess dose linearity in dermal absorption, this aspect is of relatively minor relevance.

In conclusion, our study demonstrated the feasibility of quantifying ^14^C-concentrations in plasma and microdialysates from subcutaneous adipose tissue using AMS, following the topical administration of a microdose of [^14^C]diclofenac. Unlike the dose-linear PK observed with intravenous [^14^C]diclofenac, topical dosing did not exhibit dose linearity in the rate or extent of systemic absorption. These findings add to the growing body of evidence suggesting that PK nonlinearity primarily occurs during the absorption phase (Lappin et al, 2011; van Nuland et al, 2019) and imply that microdosing may not reliably predict the disposition of certain topical drugs at therapeutic doses.

## Conflict of interest

Stephen R. Dueker is president of Sivvon Science, a company that provides AMS services to support ADME studies. Sivvon Science did not provide any financial support for this study. The other authors have no conflicts of interest to declare.
